# Confocal Laser Endomicroscopy: Real-Time Histology at the Fingertips: A Comprehensive Review of Current Applications of Endomicroscopy in Barrett Esophagus, Inflammatory Bowel Disease, and Colorectal Lesions

**DOI:** 10.3390/medicina62020415

**Published:** 2026-02-22

**Authors:** Eyad Gadour, Bogdan Miutescu, Abed Al-Lehibi, Mustafa Mohamed, Emad Aljahdli, Mohammed Albeshir, Alexandru Popa, Bodour Raheem, Antonio Facciorusso

**Affiliations:** 1 Multiorgan Transplant Centre of Excellence, Liver Transplantation Unit, King Fahad Specialist Hospital, Dammam 32252, Saudi Arabia; 2 Department of Medicine, Zamzam University College, Khartoum 11113, Sudan; 3 Department of Gastroenterology and Hepatology, “Victor Babes” University of Medicine and Pharmacy Timisoara, 300041 Timișoara, Romania; 4 Advanced Regional Endoscopy Unit, “Victor Babes” University of Medicine and Pharmacy Timisoara, 300041 Timișoara, Romania; 5 Department of Medicine, Faculty of Medicine, Al-Imam Mohammad Ibn Saud Islamic University, King Fahad Medical City, Riyadh 1223, Saudi Arabia; 6 Department of Medicine, Countess of Chester NHS Foundation Trust, Chester CH2 1UL, UK; 7 Gastroenterology Division, Faculty of Medicine, King Abdulaziz University, Jeddah 21589, Saudi Arabia; 8 Gastrointestinal Oncology Unit, King Abdulaziz University Hospital, Jeddah 22252, Saudi Arabia; 9 Section of Gastroenterology, Department of Medicine, King Fahad Specialist Hospital, Dammam 32252, Saudi Arabia; 10 Department of Gastroenterology, King Salman Medical City, Al Madinah Al Munawwarah 42319, Saudi Arabia; 11 Department of Experimental Medicine, Università del Salento, 73100 Lecce, Italy

**Keywords:** confocal laser endomicroscopy, gastrointestinal endoscopy, Barrett’s esophagus, inflammatory bowel disease, colorectal lesion

## Abstract

Confocal laser endomicroscopy (CLE) is an innovative diagnostic modality that facilitates real-time in vivo optical biopsies of various tissues within luminal and ductal structures. Since its introduction in 2004, the application of this tool has broadened from diagnostic purposes to encompass management and prognostic evaluation in fields such as gastroenterology, neurosurgery, urology, and dermatology. This comprehensive review examines the current applications of endomicroscopy in Barrett’s esophagus (BE), inflammatory bowel disease (IBD), and colorectal lesions. Evidence from the literature suggests that CLE offers a potential solution to the diagnostic limitations associated with white-light endoscopy and histology. With a diagnostic accuracy nearly equivalent to that of histology, CLE is emerging as a promising tool to mitigate the delays related to awaiting histology results for clinical and therapeutic decision-making. However, its use is mainly as a complementary diagnostic method rather than an alternative to histopathology or other ancillary studies. Nevertheless, its widespread adoption remains limited, and further research is necessary to ascertain its overall benefits and cost implications of integrating it into patient care.

## 1. Introduction

Confocal laser endomicroscopy (CLE) is an innovative advancement in gastrointestinal endoscopy that presents real-time histological visualization of living tissue, thereby functioning as an optical biopsy [1]. CLE combines the principles of confocal microscopy with those of video endoscopy. Similar to confocal microscopy, CLE employs a low-powered laser beam focused on a specific tissue plane. The fluorescence emitted from the tissues is detected and utilized to reconstruct a functional image [2]. Its application in gastrointestinal endoscopy is predicated on its anticipated higher specificity, sensitivity, and diagnostic accuracy in identifying various gastrointestinal lesions and diseases, such as atrophic gastritis lesions [2]. Besides, due to its high resolution, CLE can predict high-grade and low-grade epithelial neoplasia by analyzing gastric pit epithelial patterns [2]. The primary advantage of its utilization in guiding biopsy of various conditions lies in its higher diagnostic yield and reduced biopsy requirements compared to conventional biopsy guided by white-light endoscopy, as has been observed in the diagnosis of gastrointestinal neoplasms [2,3]. As a novel diagnostic tool, it is imperative to continually update developments in its application for diagnosing and managing different conditions. Therefore, this narrative review seeks to provide a comprehensive overview of CLE, encompassing the principles and technical details of its application in diagnosis, the advantages of its use for each condition, and its limitations.

### CLE Principles and Technology

The operational principles of CLE are based on the foundational concepts of confocal microscopy (CM), which was pioneered in 1957. Similar to CM, CLE facilitates the acquisition of real-time images of the gastrointestinal (GI) epithelia by endoscopists. These images were obtained using both standard and image-enhanced endoscopy techniques. The term “confocal” pertains to the characteristic of a system wherein the image collection mechanism and illumination are aligned on the same focal plane [4]. To fulfil this, a low-power laser beam (488 nm blue laser) is focused on a singular point. Such a singular beam of light was produced using a pinhole at the light source, with the light being reflected by a beam splitter and concentrated by an objective lens. Light from the source is focused onto a single plane of the specimen, inducing fluorescence. The fluorescence emitted by the specimen was collected using an objective lens and subsequently directed to a photodetector. The detector pinhole excludes signals from out-of-focus regions, ensuring that only light from the focal point is detected. The photodetector transmits the light impulse to a computer system that converts it into an electrical image. Ultimately, the system generates a grayscale image representing a specific plane of the epithelium [5].

## 2. Materials and Methods

A non-systematic electronic search was performed in two databases, Google Scholar and PubMed. The last search was conducted in November 2025. The search was conducted according to the various subthemes of the manuscript using the following keywords: “confocal laser endomicroscopy,” “probe-based confocal laser endomicroscopy,” “endoscopy-based confocal laser endomicroscopy,” “inflammatory bowel disease,” “Chron’s disease,” “ulcerative colitis,” “Barrett’s esophagus,” “Colorectal Neoplasms,” “colonic polyps,” “artificial intelligence,” “deep learning,” and “machine learning.” The review included all studies that provided pertinent information on the topic. Systematic reviews, meta-analyses, and randomized controlled trials were considered to be of the highest level of evidence in evaluating the efficacy of CLE in different GIT conditions.

**Basic mechanisms and components.**

**Probe-based CLE.**

Two principal CLE systems have been developed and implemented in clinical practice: probe-based CLE (pCLE) and endoscopy-based CLE (eCLE). The pCLE system consists of a laser scanning unit and a light source integrated into an external unit, with mini probes functioning as passive conduits for both light and fluorescence [4]. The fiber bundles within the pCLE mini probes are introduced into the gastrointestinal tract via the accessory channel of a standard endoscope. A variant of pCLE, the needle-based CLE (nCLE), delivers the laser beam through the needle of an endoscopic ultrasound (EUS) probe and is extensively utilized in the diagnosis of extraluminal or cystic lesions. The p-CLE system features a fixed imaging lane, with optical fibers acting as pinholes and the probe tip incorporating an optical lens. The low-powered laser emitted from the external unit was directed through the probes and objective lens to the focal point of the target tissue. The laser light is directed to a specific depth, contingent on the tissue type under examination. The tissues subsequently emit fluorescence, which is refocused by the objective lens into a light detection system within the probe [4]. There are four available p-CLE probes: ColoFlex, GastroFlex, AQ-Flex 19, and CholangioFlex [6]. A notable advantage of the p-CLE system is its ability to visualize the probe’s tip on the endoscopic image, allowing the endoscopist to view both the endoscopic and CLE visuals concurrently. This simultaneous comparison provides additional information, facilitating a more accurate assessment of the target lesion and its margins [5,7]. Table 1 outlines the characteristics of these probes.

### 2.1. Endoscope-Based CLE

In the e-CLE system, a confocal microscope is integrated into the distal end of the endoscope. The primary advantage of CLE is its capability to perform conventional endoscopy of both the upper and lower gastrointestinal tracts (GIT), with e-CLE being utilized for targeted lesions. However, a notable disadvantage is the probe’s large diameter (approximately 13 mm) and rigid end (5 cm in length). These features complicate intubation of the upper GIT. The system generates videos at a lower frame rate, specifically 0.8 frames per second (1024 × 1024 pixels) or 1.6 frames per second (1024 × 512 pixels). Nevertheless, it allows for the adjustment of the scanning depth from 0 to 250 µm within a field of view measuring 475 × 475 µm [7]. Furthermore, the system offers superior resolution, with 0.7 µm lateral and 7 µm axial, compared to the p-CLE. Nonetheless, this system is no longer commercially produced and is minimally utilized [8]. Table 2 provides a comparison between p-CLE and e-CLE features.

### 2.2. Contrast Agents and Fluorescent Dyes

To achieve tissue excitation and fluorescence generation, endoscopists employ fluorescence dyes, which are administered to patients either intravenously or through topical applications. Topical dyes used in practice include acriflavine and cresyl violet [9]. Acriflavine effectively enhances the nuclei of surface epithelial cells; however, it exhibits limited penetration into deeper tissue layers, providing minimal information about these regions [10]. Cresyl violet enhances the cytoplasm but similarly demonstrates limited tissue penetration and does not provide information on vasculature [9]. Fluorescein, the most commonly used intravenous dye, has received Food and Drug Administration (FDA) approval and is widely utilized in other fields, such as ophthalmology, with its safety well established. For patients undergoing CLE imaging, 2.5 mL of a 10% solution is administered immediately before the procedure [11]. The dye attains fluorescence within seconds of injection and can persist for up to one hour post-injection [11]. Mild adverse events may occur following injection, including diffuse erythema, rash at the injection site, transient hypotension, mild epigastric pain, and nausea and vomiting [11].

### 2.3. Advantages and Limitations Compared to Conventional Techniques

pCLE is the only CLE system currently available in the market. Its primary advantage lies in its adaptability, as it can be integrated into various scopes of different sizes and functions. This versatility has consequently expanded its application in diagnosing GIT and pancreatobiliary diseases through the probes utilized in endoscopic retrograde cholangiopancreatography (ERCP) and EUS [12]. Empirical studies indicate that p-CLE exhibits high diagnostic accuracy, with a strong correlation between p-CLE diagnoses and final histopathological outcomes [12]. CLE addresses several limitations associated with histopathology, such as increased costs, delays in obtaining results, lack of in vivo information (e.g., blood flow), and limited predictive capability regarding disease progression. Furthermore, CLE significantly reduces the number of biopsies necessary for diagnosing neoplasia [13]. In the context of endoscopy, CLE overcomes the limitation of not being able to provide a specific diagnosis regarding tissue status, whether regular or irregular [8].

## 3. Clinical Applications of Confocal Laser Endomicroscopy

### 3.1. General Clinical Utility

#### 3.1.1. Role in Real-Time Diagnosis

Since its inception, CLE has assumed a multifaceted role in the real-time diagnosis of various conditions affecting the gastrointestinal tract, pancreatobiliary system, liver, and urinary, nervous, and pulmonary systems [14,15]. Across these systems, CLE is instrumental in examining ductal structures, such as the pancreatic and bile ducts, in addition to luminal organs, including the esophagus, colon, and stomach [16]. CLE is employed to enhance endoscopic diagnoses through optical biopsies, thereby reducing the number of biopsies required for diagnosis, unnecessary resections, and follow-up procedures [16]. In the long term, this approach mitigates the risks and costs associated with numerous repetitive and indiscriminate endoscopic studies on patients [17]. The primary limitation of these systems is the lack of a standardized classification system for normal and pathological conditions across all CLE devices used in diagnosing various pathologies. The Miami consensus developed a classification of pathologies as imaged by p-CLE devices [16]. While e-CLE is not commercially available, nCLE is relatively new, and a widely adopted system for classifying diseases during its use has yet to be established. Furthermore, a single consensus and international guidelines are yet to be developed.

#### 3.1.2. Impact on Clinical Decision-Making and Patient Management

In the domain of neurosurgery, CLE has been incorporated into the surgical procedure intraoperatively. Abramov et al. [18] demonstrated that the interpretation of optical biopsies, in collaboration with pathologists through a telepathology program, achieved high accuracy in identifying underlying pathologies. This integration enables neurosurgeons to make intraoperative decisions without resorting to frozen section analysis [18]. In gastroenterology, CLE diagnosis is significantly correlated with treatment escalation within the first year of managing Crohn’s disease (CD). However, these differences are transient and diminish with extended patient follow-up [19]. Similarly, in the context of ulcerative colitis, Karstensen et al. [20] found that CLE effectively identified features indicative of disease severity, leading to treatment escalation. Moreover, this escalation in treatment resulted in significant symptom remission, which was also observable through CLE [20].

#### 3.1.3. Safety and Feasibility

The feasibility of CLE as a diagnostic tool has been established for a range of gastrointestinal (GI) conditions, including Barrett’s esophagus (BE), CD, ulcerative colitis (UC), biliary strictures, pancreatic cystic conditions, colorectal neoplasia, gastritis, gastric intestinal neoplasia, and Helicobacter pylori [17]. Safety concerns associated with CLE primarily pertain to the contrast agents and dyes employed during endomicroscopy, rather than the procedure itself. In fluorescein-based CLE procedures, significant adverse events are infrequently reported across various settings and in the extant literature. Minor adverse events are documented at a rate of approximately 1.4% of all patients [11]. An evaluation of technical safety and efficacy conducted by the American Society for Gastrointestinal Endoscopy (ASGE) and the Society of American Gastrointestinal and Endoscopic Surgeons (SAGES) Technology and Value Assessment Committee (TAVAC) has classified p-CLE as possessing an excellent safety profile in gastrointestinal endoscopy [21].

### 3.2. Confocal Laser Endomicroscopy in BE

CLE is primarily used for the surveillance and diagnosis of BE. This section highlights the pathophysiology of BE, the role of CLE in BE diagnosis, the endoscopic diagnostic criteria for BE using CLE, and the impact of CLE on BE surveillance and management.

#### 3.2.1. Pathophysiology and Clinical Significance of BE

BE is a condition characterized by metaplastic alterations in the epithelium and mucosa of the esophagus [22]. In BE, the normal squamous epithelium is supplanted by specialized intestinal columnar epithelium [22]. This metaplasia is hypothesized to arise as a complication of chronic gastroesophageal reflux disease (GERD); however, a direct causal relationship has not been definitively established, as even asymptomatic GERD patients may be affected, and the prevalence among GERD patients is only 5% [23]. The specialized metaplastic epithelium in BE is clinically significant as it predisposes individuals to esophageal adenocarcinoma [22].

#### 3.2.2. Diagnostic Challenges in BE

The diagnostic challenges associated with BE are numerous, ranging from the application of dyes in cell staining to the interpretation of results [23]. Intestinal metaplasia is the primary defining characteristic for diagnosing BE. To identify intestinal metaplasia, the presence of goblet cells within the esophageal epithelium is a key indicator [24,25]. Goblet cells are characterized by acid-mucin-filled vacuoles that distend the cellular cytoplasm, resulting in a blue stain when exposed to hematoxylin-eosin [24]. However, distinguishing goblet cells in BE from other types of glandular epithelia present in the esophagus can be challenging [24]. For example, active GERD can damage foveolar epithelial cells, causing them to accumulate acid mucin and mimic the staining of goblet cells in BE. This limitation affects the application of immunohistochemical stains, as they may yield false positives [24].

Another diagnostic challenge is the evaluation of biopsies of the gastroesophageal junction (GEJ). The associated issues may fall under the purview of either a gastroenterologist or a pathologist. The clinician is tasked with identifying suspicious lesions and determining the anatomical locations from which samples can be obtained. In contrast, the pathologist is responsible for ascertaining the presence of metaplasia in the collected samples. The precise anatomical location of the GEJ is challenging to define, as patients with GERD may exhibit irregular Z-lines or small hiatal hernias [24].

Furthermore, the criteria for defining GEJ vary among clinicians. Those utilizing endoscopic landmarks define it as the top of the rugal folds, whereas those employing histologic landmarks define it as the proximal end of the oxyntic mucosa [24]. These inconsistencies complicate the differentiation of whether the cardiac mucosa observed during histology is part of the distal esophagus (metaplasia) or the proximal stomach (normal mucosa) [24]. Additionally, chronic Helicobacter pylori infection can induce metaplasia that may be mistaken for intestinal metaplasia in BE. Therefore, biopsies must be obtained from both the GEJ and the distal stomach to assess for intestinal metaplasia in the proximal stomach [24].

#### 3.2.3. Patients with BE and Suspected Dysplasia

In patients with suspected dysplasia in BE, the final diagnosis significantly determines their management plan, whether more extensive surveillance, ablative therapy, or surgical management [24]. However, superimposed inflammatory changes observed in patients with GERD significantly affect the diagnosis of dysplasia and have demonstrated considerable interobserver variability. Such variation is highly concerning since it simply means that patients with the same degree of dysplasia may receive different therapies based on the diagnostician [24].

#### 3.2.4. Application of CLE in BE Diagnosis

Detection of dysplasia and metaplasia.

In the esophagus, p-CLE can be used to detect intestinal metaplasia, a hallmark of BE [26]. Given that intestinal metaplasia may be mistaken for cardiac mucosa depending on the anatomical location of the specimen, p-CLE offers a potential resolution to this challenge [27]. This technology enables endoscopists to conduct in vivo examinations of epithelial tissue, facilitating the assessment of the distal esophagus, GEJ, and proximal stomach within a single procedure [27].

#### 3.2.5. Criteria and Classification Systems for CLE in Barrett’s Esophagus 

The Mainz Confocal Barrett’s Classification (MCBC) was the first diagnostic criterion for BE using CLE, which was developed explicitly for e-CLE [28]. The MCBC employs the vascular and cellular architecture of both the superficial and deep mucosal epithelium to distinguish between the normal cardiac epithelium and intestinal metaplasia [28]. The normal squamous epithelium of the esophagus is characterized by scale-like flat cells with intrapapillary loops. In contrast, normal gastric mucosa exhibits a typical cobblestone appearance with regular round glands (gastric pits) and regularly shaped capillaries in the deeper mucosa [28]. Barrett’s esophagus is categorized based on the presence of either non-dysplastic (intestinal metaplasia) or dysplastic epithelium. Non-dysplastic Barrett’s esophagus is characterized by the presence of goblet cells within the columnar epithelium and mucosa, which contain dark acid mucin and exhibit regularly shaped capillaries in both the superficial and deep mucosa [28]. 

The Miami classification of BE, derived from the Mainz classification, shares similarities but was developed explicitly for p-CLE [16]. In this classification, the normal columnar epithelium is characterized by flat cells lacking villi and crypts, with bright intrapapillary loops. However, intestinal metaplasia is characterized by the presence of dark goblet cells and uniform villiform architecture within the columnar cells [29].

In addition to identifying intestinal metaplasia, p-CLE is effective in detecting neoplasia associated with BE, specifically early esophageal adenocarcinoma [30,31]. Typically, these neoplastic lesions are discovered incidentally or during routine surveillance of BE. During CLE examination, neoplastic or dysplastic epithelium is characterized by irregular and disorganized epithelial structures containing cells that appear darker than the surrounding tissue [28]. Irregularly leaking capillaries were also observed within both the superficial and deep mucosal layers [16]. 

#### 3.2.6. Comparative Studies and Diagnostic Accuracy

Kiesslich et al., who were the proponents of the MCBC, were the first to conduct an evaluation of the diagnostic accuracy of this criterion for the diagnosis of BE using e-CLE [28]. They found that the MCBC had an accuracy of 97.4% for predicting neoplasia and 96.8% for Barrett’s esophagus, with a high interobserver agreement (K value 0.84) [28]. Subsequently, Gaddam et al. developed and evaluated another criterion to identify dysplasia in BE using p-CLE [32]. This system emphasized several cellular characteristics, including gland morphology, epithelial surface characteristics, and the structure of epithelial cells. This criterion achieved an accuracy of 82% in diagnosing dysplasia, with an impressively short learning curve for endoscopists [32].

CLE has been extensively assessed in primary studies for its diagnostic utility in BE [3,31,33]. A meta-analysis encompassing seven studies, which included 3080 lesions in 345 patients, reported that p-CLE demonstrated a pooled sensitivity and specificity of 68% and 88%, respectively, in detecting adenocarcinoma [26]. Another meta-analysis, incorporating a larger dataset of 14 studies with 4047 lesions in 789 patients, revealed a higher pooled sensitivity of 89% and a lower specificity of 83% for CLE. Such outcomes suggested a variability in the diagnostic accuracy of e-CLE with increased sample sizes [34]. This analysis also indicated that p-CLE exhibited superior diagnostic accuracy compared to e-CLE [34]. The latest systematic review and meta-analysis revealed that incorporation of p-CLE as an adjunct to four-quadrant biopsies for BE resulted in a 5% increase in absolute detection of neoplasia in these patients [35].

#### 3.2.7. Impact on Surveillance and Treatment Strategies

As CLE facilitates real-time histological examination of the mucosal layer in BE, it can significantly inform decisions regarding subsequent surveillance and patient management. CLE has demonstrated high diagnostic accuracy in identifying metaplasia and neoplasia in patients with Barrett’s esophagus [34]. Consequently, CLE enables endoscopists to make real-time determinations about the necessity for further endoscopic evaluation or surgical resection of lesions, with minimal reliance on histological analysis [28]. However, despite the evaluated diagnostic accuracy of CLE in patients, its application as a standalone decision-making tool in the management of BE remains underutilized, warranting further investigation. 

The latest endoscopy guidelines currently recommend against the routine use of CLE with the Seattle protocol in BE surveillance. The recommendation has been attributed to the inconsistent and low-quality evidence on the net benefit of CLE to the patients [36]. Moreover, the observed benefit is currently small at best. Hence, when weighed against the associated costs, training, and risk associated with CLE, it does not warrant its incorporation into routine surveillance [25]. Moreover, other guidelines on the diagnosis and management of BE by the British Society of Gastroenterology and American College of Gastroenterology (ACG) have yet to consider CLE use in the diagnosis and management of BE [37,38].

#### 3.2.8. Confocal Laser Endomicroscopy in IBD

This section highlights the application of CLE in IBD. In the process, the section first identifies the pitfalls in IBD diagnosis, highlights the role of CLE in addressing these pitfalls, and discusses the impact of CLE on the management of IBD.

#### 3.2.9. Overview of IBD (Crohn’s Disease and Ulcerative Colitis)

IBD is characterized by chronic inflammation of the intestines, which is predominantly idiopathic. Clinically, IBD is categorized into two distinct conditions: Crohn’s disease (CD) and ulcerative colitis (UC). This classification is based on the disease location, clinical manifestations, and histopathological features. CD is typically a transmural condition that can affect any segment of the GIT and is marked by granuloma formation. In contrast, UC is confined to the colon and results in superficial ulcerative disease accompanied by persistent inflammation [39]. IBD is frequently associated with other severe conditions, such as colorectal cancer and primary sclerosing cholangitis [40]. It is most commonly diagnosed in individuals between the ages of 20 and 40 years [41].

#### 3.2.10. Challenges in IBD Diagnosis and Assessment

The diagnosis of IBD is predicated upon the results of a variety of assessments, including endoscopic, clinical, radiological, and histological evaluations [42]. The initial patient evaluation involves a comprehensive history to identify recent exposures, medication history, infectious diseases, and other risk factors associated with IBD. The primary clinical manifestations in patients encompass abdominal pain, palpable masses indicative of intra-abdominal abscesses or phlegmon, and various perianal alterations, such as fissures and fistulas [43]. Laboratory evaluation of patients is generally routine and often reveals mild anemia and thrombocytosis. Furthermore, stool analyses are instrumental in excluding infectious etiologies of the observed symptoms. Additional diagnostic procedures include endoscopy, magnetic resonance enterography (MRE), enteroscopy, and esophagogastroduodenoscopy [43]. Numerous challenges are encountered in the diagnosis of IBD, predominantly in the histopathological examination of specimens. These challenges are primarily categorized into three areas: differentiating IBD from other mimicking diseases, distinguishing between CD and UC, and diagnosing disease complications while assessing the involvement of various segments of the GIT [44]. Several diseases mimic IBD, including intestinal tuberculosis, Behçet’s disease, infectious colitis, and other miscellaneous conditions [45]. Due to the similarities among these conditions, misdiagnosis may lead to inappropriate treatment, adversely affecting the patient’s remission [45].

#### 3.2.11. Role of CLE in IBD

##### Detection of Microscopic Inflammation

Microscopic examination and interpretation of biopsies are essential for the diagnosis and management of IBD. Histological findings contribute to diagnosis, subclassification, and assessment of drug efficacy, as well as the detection of disease complications [46]. The presence of microscopic inflammation and disease activity serves as a predictor of disease relapse, whereas its absence indicates potential disease remission [47]. The European Society of Pathology and the European Crohn’s and Colitis Organization (ECCO) recommend that during diagnostic endoscopy, two biopsies should be obtained from each of at least five or six segments of the bowel, including the rectum and terminal ileum [48,49]. This guideline does not apply to patients undergoing treatment.

Furthermore, many gastroenterologists continue to perform extensive sampling of affected bowel areas while avoiding unaffected regions. This practice may underestimate disease severity, particularly when employing disease scoring methods that consider the number of affected areas [50]. CLE can be used to investigate microscopic activity in patients with IBD. Neuman et al. utilized CLE to evaluate microscopic activity in patients with CD, demonstrating that CLE possesses high sensitivity and accuracy in identifying microscopic disease in patients with CD. The authors further proposed a Crohn’s Disease Endomicroscopic Activity Score (CDEAS) for assessing disease activity in vivo [51].

#### 3.2.12. CLE in Differentiating IBD Types and Identifying Dysplasia

The differential diagnosis between CD and UC is of significant clinical importance because the management strategies for these IBD entities are distinct [52]. This differential diagnosis is based on a comprehensive clinical evaluation of the patient, along with histological, endoscopic, biochemical, and radiological assessments. Nevertheless, the classification of IBD as unclassified remains prevalent in approximately 3–6% of adult patients during their initial evaluation. CLE, which facilitates in vivo histology through optical biopsies, has been proposed as an adjunct method for the histologic characterization of CD or UC [49,52]. 

Tontini et al. pioneered the use of CLE in the differential diagnosis of UC and CD, developing a CLE scoring system known as the IBD Differentiation based on the Endomicroscopic Assessment (IDEA) scoring system [53]. The IDEA score system is a point system in which more points are awarded if the patient has CLE findings commonly associated with UC and lacks the CLE findings associated with CD [53]. A diagnosis of UC is then made if the patient has ≥ 6 total points, while a diagnosis of CD is made if the patient has < 6 total points (Table 3). In the pilot study, the IDEA score had an accuracy of 93.7% in differentiating between CD and UC [53]. 

In the context of IBD diagnosis, CLE can also be employed to identify dysplasia in patients with IBD. Rispo et al. assessed the diagnostic accuracy of CLE in detecting dysplasia in UC and found that CLE could identify dysplasia with a sensitivity of 100% and specificity of 90% [54]. Lord et al. also compared CLE to other optical chromoendoscopy techniques for characterizing lesions in IBD and thus differentiating between neoplastic and non-neoplastic lesions [55]. They found that real-time CLE had the highest pooled sensitivity and specificity (91% and 97%, respectively) compared to magnification endoscopy (90% and 87%), virtual chromoendoscopy (86% and 87%), and dye-based chromoendoscopy (67% and 86%), respectively [55]. Given this level of diagnostic precision, CLE presents a viable alternative and feasible option to reduce the number of random biopsies required for the surveillance of dysplasia in IBD, particularly in UC [54]. However, the net benefit of CLE on IBD surveillance is yet to be determined.

#### 3.2.13. Effect on Therapeutic Monitoring and Prognosis

Evaluating prognostic factors in IBD is crucial, as it facilitates early intervention and the implementation of treat-to-target strategies [19]. CLE can be employed to characterize mucosal inflammation in IBD, thereby predicting the clinical outcomes in affected patients. In a prospective study, Tontini et al. assessed the utility of CLE in predicting clinical outcomes in patients with CD by examining two histological markers: crypt architectural abnormality and focal cryptitis [19]. Their findings indicated that CLE could reveal clinical features of mucosal inflammation, thus predicting medical escalation within one year and the development of transmural lesions [19].

CLE is also used to assess local barrier dysfunction, a marker of disease activity, and symptom remission. Kiesslich et al. [56] employed CLE to evaluate epithelial shedding in IBD as an indicator of local barrier dysfunction. Their study demonstrated that CLE could detect cell shedding in patients in clinical remission, with increased cell shedding being associated with relapse within one year of endoscopic examination [56]. The accuracy of this method in predicting relapse was reported to be 79% [56]. Karstensen et al. [57] found that ileal fluorescein leakage and microerosions were markers for predicting relapse in CD. These two features were significantly associated with relapse within a year in both the patients in remission and those with endoscopically active disease [57].

#### 3.2.14. Confocal Laser Endomicroscopy of Colorectal Lesions

This section focuses on colorectal lesions, including benign colorectal polyps and colorectal cancer (CRC). This section provides an overview of colorectal lesions and the importance of their detection. This section further explores how CLE addresses some of the challenges observed in colorectal lesion detection. Finally, this section highlights the impact of CLE on the management of colorectal lesions.

### 3.3. Epidemiology and Importance of Colorectal Lesions Detection

Colorectal polyps are relatively prevalent, particularly among older adults, with an estimated prevalence ranging from 30% to 50% [58]. A well-established association exists between colorectal polyps and the development of CRC [59]. However, it is crucial to recognize that the majority of these polyps or lesions are benign and do not progress to CRC. In cases where progression to CRC occurs, it typically takes over a decade and follows three distinct pathways [60]: the Chromosomal Instability Pathway (70%), the Microsatellite Instability Pathway (15%), and the Serrated Pathway (15%) [61]. Among colorectal polyps, adenomas are the most common, accounting for up to 60% of all polyps, followed by hyperplastic polyps (up to 30%) and sessile serrated lesions (up to 18%) [61]. Conversely, CRC is a highly prevalent form of cancer and ranks as the second leading cause of cancer-related mortality worldwide, with a lifetime morbidity risk of 4% to 5% and a lifetime mortality risk of 3.2% to 3.4% [62]. Fortunately, the progression of CRC from colorectal polyps is protracted, allowing for early detection, rendering CRC a preventable condition [62]. The US Preventive Services Task Force recommends CRC screening for all adults aged 50 to 75 years. Screening modalities include computed tomography (CT) colonography or flexible sigmoidoscopy every five years, or colonoscopy every ten years [62]. The objective of this screening is to identify precursor lesions and resect or treat them before they progress to CRC [63]. Landmark studies have demonstrated that an increase in adenoma detection rates correlates with a 37% reduction in the risk of interval cancer and a 50% reduction in CRC-related mortality [63]. This benefit is even more pronounced when screening is conducted by experienced colonoscopists, with a 73% reduction in the risk of interval cancer and an 82% decrease in CRC-related mortality [63].

#### 3.3.1. Application of CLE in Colorectal Adenomas and Early Cancer

Kiesslich et al. were pioneers in reporting the application of CLE for the in vivo detection of intraepithelial neoplasia in CRC [64]. Their pilot study demonstrated that CLE could predict neoplastic changes with a high accuracy of 99.2% [64]. In a separate investigation, de Palma et al. examined 20 adults with 32 polyps and established that p-CLE predicted adenomatous histology with a negative predictive value and sensitivity of 100%, and a specificity of 85% [65]. Furthermore, p-CLE exhibited higher sensitivity compared to virtual chromoendoscopy (91% versus 77%), while maintaining comparable specificity (76% versus 71%) [66]. A meta-analysis by Su et al. found that real-time CLE results in higher specificity (97% vs. 82%; *p* < 0.001) and sensitivity (96% vs. 8%%; *p* < 0.001) than blinded CLE [67]. Moreover, the review found that in real-time CLE, e-CLE systems had better specificity (99% vs. 82%; *p* < 0.0001) and sensitivity (96% vs. 89%; *p* < 0.0001) than p-CLE systems [67].

#### 3.3.2. Comparative Effectiveness Versus Other Imaging Modalities Like Narrow Band Imaging and Chromoendoscopy

Numerous studies have assessed the comparative effectiveness of CLE in distinguishing between neoplastic and non-neoplastic colorectal lesions. Kuiper et al. conducted a comparison between chromoendoscopy and p-CLE, revealing that chromoendoscopy exhibited superior diagnostic accuracy compared to CLE (89.3% versus 71.9%) [68]. Conversely, Buchner et al. reported that p-CLE demonstrated higher sensitivity (91% vs. 77%, *p* = 0.10) and comparable specificity (76% vs. 71%, *p* = 0.77) relative to chromoendoscopy in differentiating neoplastic from non-neoplastic lesions [66]. Similarly, their findings indicated that p-CLE had greater sensitivity than Fujinon intelligent color enhancement (FICE) (93% vs. 73%, *p* = 0.003) and nearly equivalent specificity (86% vs. 68%, *p* = 0.22). However, no significant difference was observed in the sensitivity (88% versus 84%, *p* = 1.00) and specificity (63% versus 75%, *p* = 0.68) between p-CLE and narrow-band imaging (NBI), respectively [66]. In contrast, Shahid et al. found that p-CLE exhibited higher sensitivity than NBI (86% versus 64%), while NBI demonstrated greater specificity than p-CLE (92% versus 78%) [69]. 

#### 3.3.3. CLE Criteria for Malignancy Assessment

Two studies have established objective criteria for distinguishing between neoplastic and non-neoplastic colorectal lesions using CLE [64,66]. Certain studies have employed the Mainz criteria, which were initially proposed for the general differentiation of neoplastic and non-neoplastic lesions within the GIT [64,66]. Subsequently, Sandulenanu developed the adenoma dysplasia score system (ADS) to differentiate high-grade dysplasia from low-grade dysplasia. This system assigns an adenoma dysplasia score ranging from 0 to 2 based on four adenoma characteristics: epithelial surface maturation, crypt architecture, vascular pattern, and cytonuclear atypia [70]. Later, Xie et al. introduced another scoring system to distinguish between non-adenomatous and adenomatous polyps, which is a modified version of the Mainz criteria [71]. This criterion classifies adenomatous lesions as those exhibiting any of the following features: goblet cell depletion, villous architecture, or microvascular alterations [71]. The criterion demonstrates high diagnostic accuracy (95%) and interobserver agreement (kappa = 0.93) [71]. Table 4 gives a summary of the colorectal polyps classification system.

#### 3.3.4. Role in Endoscopic Resection and Margin Assessment

Residual neoplasia following endoscopic mucosal resection (EMR) of colorectal polyps is a prevalent issue [72]. The assessment of this residual neoplasia using conventional endoscopy remains challenging, thereby necessitating the use of CLE [73]. Shahid et al. [72] investigated the efficacy of p-CLE in comparison to virtual chromoendoscopy for detecting residual neoplasia post-EMR. In a cohort of 92 patients with 129 EMR scars, they reported a sensitivity, specificity, positive predictive value (PPV), negative predictive value (NPV), and accuracy of 97%, 77%, 55%, 99%, and 81%, respectively [72]. No additional studies have been conducted on this topic, underscoring the limited evidence available to adequately assess the utility of p-CLE.

#### 3.3.5. Training in CLE

Endoscopists are commonly trained in CLE didactically or through online resources. Didactic training led by expert endoscopists has been shown to produce rapid learning curves for the diagnosis and interpretation of various GI diseases using CLE [74,75,76,77]. Self-directed audiovisual learning with online resources has been proposed as a cost-effective approach to CLE training, in which learners have control over their learning approach. However, evidence indicates that didactic learning results in higher overall accuracy, sensitivity, and interobserver agreement compared to self-directed learning for CLE [74]. The required dataset for CLE training in GI endoscopy varies from study to study. Standard image groups include six sets of 30 images [74,78], one set of 20 images and videos [75,76,77], and one set of 10 images [77]. The best choice of training dataset is yet to be determined, and each of the evaluated studies only utilized one imaging set. 

The learning curve for CLE has been shown to be a steep one. Experienced endoscopists have higher accuracy than inexperienced endoscopists in the initial stages of training, but the difference narrows upon completion of their training [78,79]. Moreover, there is no significant difference in the skill acquisition between inexperienced endoscopists and pathologists, indicating that previous pathology knowledge does not impact learning for CLE [78]. Additionally, it has been observed that expert endoscopists and expert GI pathologists use different approaches to interpret CLE images, resulting in poor interobserver agreement between the two [76]. The confidence of the endoscopists and image quality are positive predictors for the accuracy of endoscopists during training [74,75,78]. These two factors may partly explain why expert endoscopists perform better in earlier training compared to the other analysts, since they are more confident and able to obtain higher quality images [79].

#### 3.3.6. Patient Perspective and Procedural Logistics

Few studies have investigated the time reduction from incorporating CLE into gastrointestinal endoscopy. In neurosurgery, Wagner et al. found that CLE had a significantly lower assessment time compared to frozen sections (3 min versus 27 min, *p* < 0.001). The study demonstrated that CLE could significantly reduce assessment time without significantly compromising the diagnostic accuracy since CLE achieved a diagnostic accuracy of 0.87 compared to 0.91 of frozen section, with the difference not statistically significant (*p* = 0.367) [80]. In GIT, Li et al. compared CLE to pCLE in terms of procedural duration and accuracy. They found that the procedural time of pCLE was significantly shorter than that of eCLE during colonoscopy (32.48 versus 39.89, *p* < 0.001) and during EGD (16.78 versus 18.13 min, *p* = 0.27), with comparable diagnostic accuracy [81].

Patient cooperation is key during CLE in both upper and lower GI CLE. Using sedation during CLE can improve patient cooperation and the quality of CLE. Chu et al found that sedating patients during CLE significantly improves the diagnostic accuracy of CLE compared to the lack of sedation [82]. The sedation during CLE can be achieved using different agents, including propofol, midazolam, and fentanyl. Kiesslicch et al. utilised propofol for conscious sedation during colonoscopy [56]. In upper GIE, Zuo et al. found that sedation using propofol during CLE resulted in significantly more good quality images and significantly lesser duration and sedation times compared to sedation using midazolam plus fentanyl [83]. Sedation during CLE can also affect patient comfort. Chu et al. found that the choice of sedating agent did not have a significant effect on patient satisfaction during the procedure [82]. However, compared to repeated biopsies, the reduced number of biopsies due to integration of CLE significantly reduces patient discomfort during the diagnostics [84]. This, therefore, increased the patient’s satisfaction during the procedure [84].

#### 3.3.7. Regulatory Approval of CLE

CLE systems and related contrast agents, such as fluorescein dye, have received regulatory approvals, such as FDA clearance and CE marking. The CLE systems are cleared using a 510 (k) substantial equivalence process since they are classified as class II devices, as they are a modification of confocal endomicroscopy [85]. To date, only two CLE systems have received FDA clearance. The first system to be cleared was the F-600 system in 2006, which was cleared for internal imaging of tissue microstructure. The F600 system can be used with any standard endoscope with a working channel of at least 2.8 mm [86]. The second CLE system to be cleared was the Cellvizio^®^ 100 series system (Mauna Kea Technologies, Paris, France) with Confocal Miniprobes^TM^, which is a probe-based system [87]. The cleared probes for the GIT include the GastroFlex and ColoFlex [87]. Across the systems, the dyes that have been cleared for use in CLE include fluorescein sodium (IV) and pafolacianine. 

#### 3.3.8. Current Challenges and Limitations of CLE

Despite the high accuracy of CLE, its clinical application remains constrained by various barriers and inherent limitations. Factors impeding the utilization of CLE include the absence of insurance reimbursement for CLE procedures, lack of established protocols for CLE indications, insufficient technical expertise for interpreting CLE images, and the cost associated with the procedure [12]. CLE also has various technical limitations. A primary limitation of this technique is its inability to provide the tissue or specimens necessary for molecular characterization. Molecular diagnosis is crucial for clinicians to classify tumors and identify therapeutic and prognostic markers. Consequently, while CLE may identify poorly differentiated tumors, it fails to furnish additional information that could guide patient care [13]. Molecular imaging using labeled peptides has been proposed as a potential solution to this limitation; however, the development of such peptides is time-consuming and subject to rigorous scrutiny by the FDA due to the underlying mechanisms of these particles [13].

Furthermore, CLE does not penetrate the gastrointestinal mucosa, thereby providing limited information for the diagnosis of tumor mucosal invasion. Consequently, concurrent mucosal resection, which could expedite the transition from diagnosis to treatment, is not feasible [13]. A theoretical solution involves the use of fine needle-sized probes; however, these probes do not offer sufficient additional benefits to justify their use over other minimally invasive micro-biopsy techniques, thus impeding the development of such technology. Another limitation of CLE is that, while the current evidence shows promising results, various aspects of its use, including cost and practical implications, have yet to be widely investigated. Since most of the studies are conducted in academic centers, the lack of such data further hinders the translation of these findings to non-academic real-world centers in which CLE is to be integrated into patient care. 

Economic factors, including capital acquisition costs, contrast agent-related expenses, ongoing maintenance, and training, significantly influence CLE’s adoption of CLE in clinical practice. The evidence on the cost implications of CLE is still limited. The initial cost of the equipment has been approximated to be $150,000 with an additional $500 to $800 expense for disposable probes used with pCLE systems [87]. The high cost associated with the use of CLE has been cited as one of the barriers to the adoption of CLE in clinical practice, especially in low-prevalence settings [17]. Saumoy et al. analysed the cost-effectiveness of using the Seattle biopsy protocol versus optical coherence tomography (OCT) or CLE in BE surveillance. They found that CLE increased the cost of surveillance by $1983 compared to the Seattle protocol, but was less effective in diagnosis (−0.00018) [88]. Various studies have stated that reimbursement for CLE is limited [87]. Moreover, since the available scarce evidence indicated that CLE may not be more cost-effective than standard biopsy protocols, some policies have cited not covering the associated costs of CLE due to a lack of sufficient data on its net benefit. However, no peer-reviewed study has been published that evaluated the reimbursement rates and challenges for CLE procedures.

#### 3.3.9. Future Directions and Innovations

##### Integration with Artificial Intelligence and Machine Learning

Machine learning (ML) has been proposed as a method to enhance the detection of lesions using CLE in the GIT [89]. ML models improve diagnostic accuracy by recognizing patterns that may have been missed by human visual analysis [90]. Computer-aided diagnosis (CAD) has been employed for diagnosing colorectal adenocarcinoma with CLE, achieving an overall diagnostic accuracy of 85% [89]. In the evaluation of colonic polyps, the integration of CAD significantly enhances the diagnostic accuracy of CLE to 94% [91]. Pulido et al. developed convolutional neural networks (CNN) for differentiating dysplasia, metaplasia, and neoplasia of p-CLE images of the oesophagus [92]. The best model developed had an F1 score of 0.89 and differentiated p-CLE visuals with these three features, with a specificity of 0.90 and a sensitivity of 0.88 [92]. 

CNNs, such as the one utilized by Pulido et al., are the most suitable deep learning techniques for analyzing the image data generated during endoscopy. Models developed using CNN outperform models developed from other traditional ML techniques in tasks such as image recognition, segmentation, and classification [93,94]. Moreover, unlike conventional ML, CNN can also be applied to dynamic video streams, such as those in real-time video streams, making it more suitable for GI endoscopy [95]. While none of the video dynamic AI models have been used in the diagnosis of the three conditions discussed in this review, their feasibility has already been demonstrated in gastric imaging. Zheng et al. [96] developed a system that conducted real-time annotation of p-CLE detection areas from videos. This system achieved an accuracy of 96% in the identification of gastric intestinal metaplasia [96]. 

The initial findings from these pilot studies of AI in GI endoscopy and CLE indicate a promising trend in which the integration of AI will improve the diagnostic accuracy of endoscopists. Moreover, these systems will be able to alert endoscopists to possible suspicious lesions that would have otherwise been overlooked in their analysis. This may make the clinician more observant and detailed in their image analysis, resulting in a better and well-informed diagnosis. However, this application of CLE remains under-researched and requires further investigation. Some studies have utilized CAD in conjunction with volumetric laser endomicroscopy, representing another potential advancement [97].

### 3.4. Potential Expanded Indications

CLE plays an established role in the diagnosis of gastrointestinal disorders. Its application extends to ophthalmology, urology, and neurosurgery [15]. In the field of neurosurgery, CLE is integrated intraoperatively for tumor resection [15]. This technique has also been explored in general surgical procedures, demonstrating promising safety and feasibility [98]. However, the role of CLE in providing intraoperative guidance for lesion resection requires further investigation. Additional research in this area could establish CLE as an invaluable tool, particularly in serial resection surgeries that necessitate margin assessment [99].

While CLE provides real-time optical biopsy capabilities, the integration of AI holds promise for further enhancing diagnostic accuracy by reducing operator dependency and improving lesion detection through automated image analysis. AI-assisted CLE systems, particularly those employing convolutional neural networks, may augment endoscopists’ interpretations by recognizing subtle histologic patterns, potentially accelerating learning curves and standardizing assessments. However, whether AI might entirely supersede CLE or serve primarily as a complementary tool remains to be elucidated, pending further clinical validation. Economic factors, including capital acquisition costs, contrast agent-related expenses, ongoing maintenance, and training, significantly influence CLE’s adoption of CLE in clinical practice. Some studies suggest potential cost savings through reduced biopsy numbers and improved diagnostic efficiency; however, comprehensive cost-effectiveness and budget-impact analyses remain limited.

#### 3.4.1. Ongoing Clinical Trials and Research Trends

Current research on the utility of CLE in patients with Barrett’s esophagus is lacking. In the context of IBD, the clinical trial NCT07121920 is investigating the integration of proteomic profiling to evaluate the treatment response. Another study, NCT06624579, aimed to elucidate the prognostic value of CLE in predicting treatment outcomes in UC. Additionally, certain studies, such as NCT06505304, aim to evaluate the utility of AI-enhanced CLE in predicting postoperative relapse in CD. Other investigations, including NCT06505304, focus on establishing the efficacy of CLE in assessing surgical margins during CD resection, potentially reducing recurrence rates. In patients with colorectal cancer, trial NCT05879783 was designed to evaluate the utility of CLE in identifying peritoneal metastasis of CRC in vivo during exploratory laparoscopic surgery.

#### 3.4.2. Limitations of the Narrative Review

There are various limitations to our narrative review. One of the main limitations of our study is the non-systematic literature search and inclusion of most of the relevant studies without a specific inclusion criterion. The reported findings in the study are therefore based on a heterogeneous set of studies. Since the diagnostic accuracy of CLE can be affected by different factors inherent to the study designs, our findings are not conclusive but descriptive. Interpretation of the findings must therefore be done with caution. Additionally, the included studies are mostly small pilot studies investigating different aspects of the use of CLE and with varying inclusion criteria of patients. This, therefore, partly explains the discrepancies across trials, particularly comparing CLE with chromoendoscopy, underscoring the need for standardized protocols and larger multicenter trials to evaluate CLE’s clinical performance robustly.

## 4. Conclusions

This narrative review demonstrated that the application of CLE in the diagnosis and management of BE, IBD, and CRC has grown significantly over the past two decades. As a real-time in vivo optical biopsy tool, CLE offers potential solutions to the limitations inherent in conventional endoscopy and histology. However, CLE does not provide an alternative to these diagnostic methods but rather a complementary diagnostic method to improve them. However, certain limitations still hinder its application, necessitating further investigation.

Despite these advances, significant gaps remain in the translation of CLE into routine clinical practice, including the absence of universally accepted diagnostic criteria, standardized training protocols, and insurance reimbursement models. Moreover, the clinical utility of CLE in guiding treatment decisions, long-term outcomes, and cost-effectiveness requires further investigation. Integration with AI and emerging minimally invasive platforms presents exciting avenues; however, comprehensive comparative effectiveness research is necessary to define optimal deployment strategies.

## Figures and Tables

**Table 1:** Characteristics of the different p-CLE probes.

Characteristics	CholangioFlex	GastroFlex UHD	ColoFlex UHD	AQ-Flex 19
Compatible procedures	Endoscopic retrograde cholangiopancreatography (ERCP).	Upper endoscopy	Colonoscopy	EUS-based fine needle aspiration (EUS-FNA)
Indications	Intermediate pancreatic and biliary strictures.	Barrett’s Esophagus (BE), gastric neoplasia.	Colorectal polyps.	Cystic lesions of the pancreas.
Compatible channel	≥1.2 mm	≥2.8 mm	≥2.8 mm	≥0.91 mm (19-gauge) FNA needle.
Field of view diameter (µm)	320	240	240	325
Probe length (m)	4	3	3	4
Image resolution (µM)	3.5	1	1	3.5
Confocal depth (µm)	40 to 70	55 to 65	55 to 65	40 to 70
Maximal number of uses	10	20	20	10

Note: CholangioFlex, GastroFlex UHD, ColoFlex UHD, and AQ-Flex 19 are registered trademarks.

**Table 2:** Comparison of p-CLE and e-CLE.

Characteristic	e-CLE	p-CLE
Location of the CLE probe	Integrated into the endoscope’s tip	Goes through the endoscope’s accessory channel
Image acquisition	Lower compared to p-CLE	Higher compared to e-CLE
Field of view	Larger compared to p-CLE	Smaller compared to e-CLE
Resolution	Better compared to p-CLE	Poorer compared to e-CLE
Imaging depth	Adjustable	Fixed

**Table 3:** The IDEA scoring system for differentiating between UC and CD [53].

CLE Findings	Presence	Absence
Severe and widespread architectural distortion	3	0
Frankly irregular surface	3	0
Decreased crypt density	3	0
Discontinuous crypts architectural abnormality	0	1
Focal cryptitis	0	1
Discontinuous inflammation	0	1

**Table 4:** A colorectal polyps classification system (the Adenoma dysplasia score).

Feature	LGD	HGD
**Epithelial surface maturation**	0–1	1–2
0: normal		
1: incomplete maturation		
2: lack of epithelial surface maturation		
**Crypt architecture**	0–1	1–2
0: normal		
1: enlarged, slightly crowded crypts		
2: crowding, distorted crypts		
**Vascular pattern**	0–1	1–2
0: normal		
1: slightly increased vascular pattern, preserved hexagonal pattern		
2: increased, distorted vessels		
**Cytonuclear atypia**	1	2
0: basal, regular nuclei		
1: pseudo-stratification of regular, pencillate nuclei		
2: pseudo-stratification of irregular, large, round, more apically localized nuclei		
**Adenoma dysplasia score (ADS)**	**1–4**	**5–8**

## Data Availability

The raw data supporting the conclusions of this article will be made available by the authors on request.

## Abbreviations

AI	Artificial Intelligence
ACG	American College of Gastroenterology
ADS	Adenoma Dysplasia Score
BE	Barrett’s Esophagus
CDEAS	Crohn’s Disease Endomicroscopic Activity Score
CD	Crohn’s Disease
CE	Conformité Européenne (European Conformity Marking)
CLE	Confocal Laser Endomicroscopy
CM	Confocal Microscopy
CRC	Colorectal Cancer
CT	Computed Tomography
ECCO	European Crohn’s and Colitis Organization
EMR	Endoscopic Mucosal Resection
ERCP	Endoscopic Retrograde Cholangiopancreatography
FDA	Food and Drug Administration
FICE	Fujinon Intelligent Color Enhancement
GI	Gastrointestinal
GIT	Gastrointestinal Tract
GERD	Gastroesophageal Reflux Disease
GEJ	Gastroesophageal Junction
IDEAS	IBD Differentiation based on the Endomicroscopic Assessment
IBD	Inflammatory Bowel Disease
MCBC	Mainz Confocal Barrett’s Classification
MRE	Magnetic Resonance Enterography
ML	Machine Learning
nCLE	Needle-based Confocal Laser Endomicroscopy
NBI	Narrow Band Imaging
pCLE	Probe-based Confocal Laser Endomicroscopy
PPV	Positive Predictive Value
NPV	Negative Predictive Value
UC	Ulcerative Colitis
US	United States
